# Consumer Information Needs and Sensory Label Design for Fresh Fruit Packaging. An Exploratory Study in Spain

**DOI:** 10.3390/foods10010072

**Published:** 2020-12-31

**Authors:** Paula Fernández-Serrano, Paula Tarancón, Cristina Besada

**Affiliations:** Sensory and Consumer Science Research Group, Postharvest Technology Center, Valencian Institute for Agricultural Research (IVIA), Carretera Moncada-Náquera, km. 4.5, 46113 Moncada, Valencia, Spain; fernandez-serrano_pau@externos.gva.es (P.F.-S.); tarancon_pau@gva.es (P.T.)

**Keywords:** consumer, information gaps, packaged fruit, sensory label, purchase decision

## Abstract

In recent decades, packaged fruit has gained market ground over loose fruit, and fruit containers have become a source of information for consumers. This study approaches three objectives related to consumer information needs for decision making when purchasing fruit: (1) Determine if consumers’ choice of packaged fruit rather than loose fruit is motivated by their interest in the information provided on packaging; (2) identify information gaps on fruit packaging labels; (3) identify those sensory attributes that consumers consider to be of major interest to be included in sensory labels of different fruit types. The study was based on an online questionnaire answered by 394 fruit consumers. Ninety percent of the participants stated having an interest in receiving information when purchasing fruit, but their choice between packaged or loose fruit was not conditioned by their information needs. Moreover, a gap between information interest and information use was detected as their final purchase decisions were not always based on the provided information. ‘Harvest date’, ‘production method’, ‘percentage of the price received by the farmer’, ‘applied treatments’, ‘sensory characteristics’, and ‘environmental information’ were identified as the major information gaps, as these labels were unavailable for a high percentage of consumers, who stated their interest in them. According to consumers, sensory labels should include information about ‘sweetness’ and ‘flavor intensity’ irrespectively of fruit type. ‘Sourness’ and ‘juiciness’ attributes were particularly interesting for citrus, as ‘sourness’ and ‘firmness’ were for kiwi. Information about texture properties was required for pome and stone fruit. Other attributes, such as easiness to peel, were important only for citrus fruit.

## 1. Introduction

In most occidental countries, the fruit market has undergone major changes in the last 50 years, and has moved from selling local production to commercializing fruit imported from countries worldwide. This change in fruit marketing has led supply to sharply increase, which is linked not only with the availability of off-season fruits, but also with the possibility of choosing among a wider range of varieties [1,2,3,4]. Moreover, in the last few decades, production systems that respect the environment and health (organic, bio, etc.) have been implemented, and the production method is now another factor that broadens consumer choice options [5,6]. In light of such an offer, information about the fruit provided to consumers at points of sale has become a key factor for their purchase decisions [7].

In parallel to this increasing offer, the way of commercializing fruit has also changed in recent decades. Thus, packaged fruit has gained market ground and presently co-exists with loose fruit on supermarket shelves. Apart from preserving fruit from mechanical damage, packaging has been claimed to extend the fresh fruit shelf life by prolonging freshness and food quality, and ensuring safety [8,9]. However, the main handicap of fruit and other products’ packaging is a negative environmental impact, such as plastic overuse. Thus, reducing plastic packaging has become one of the main challenges of industry in the last few years [10].

Traditionally, loose fruit consumers have received fruit information orally when asking grocery sellers, or in writing in texts added to labels on shelves together with prices, or in fixtures or posters in supermarkets [11]. However, while supermarkets have gained ground compared to small groceries, packaged fruit has gained ground over loose fruit, and written information has become the main channel to inform consumers. With packaged fruit, as with other foodstuffs, containers and packaging are a channel that provides consumers with more information than that displayed only on shelf labels [12,13]. As a result, consumers now receive much more information when they purchase packaged fruit than when they buy loose fruit. Thus, package labels can be defined as any information printed on food packaging, including nutrition information panels, use and storage information, nutrient content claims, health claims, country of origin labelling, as well as labels with organic production or sustainably produced data, etc. [14,15]. 

Therefore, to a certain extent, information has become part of the fruit market offer. Factors like label information source [16,17], label design, or label combinations [14], among others, have been reported to influence consumer trust in packaged label information. According to Rupprecht et al. (2020) [17], a common refrain in such research points out the large volume and diversity of labels on the market today, and how this makes it difficult for consumers to know who provides information and whether it is trustworthy. 

Therefore, it is possible that consumers’ choice between loose and packaged fruit is conditioned by their information needs for purchase decisions and/or their trust in the provided information. Despite the relevance that clarifying this aspect may have for industry, to our knowledge this has not yet been investigated.

However, different studies have elucidated the effect of specific label facts and packaging claims on consumer decisions when choosing among packaged fruit [5]. Adding facts on fruit packaging about specific credence attributes, such as production methods [18,19], country of origin [6,20], nutritional claims [21,22], or even packaging characteristics [23], has been shown to impact fruit choice. However, fruit packaging usually contains claims about not only one of these aspects, but many of them. Several studies support the notion that not all information is read, and that too much information can lead to consumers not assimilating it all due to rush buying or time pressure [13,20,24]. Navigating a sea of food labels can cause “label fatigue” and undermine the intention of providing information in the first place [25,26]. Therefore, it is important to investigate consumer responses to the information contained on fruit packages from a wider perspective to identify the facts that consumers are really interested in and those that are secondary. In line with this, it is also necessary to identify information gaps, i.e., information that consumers need for making a fully-informed decision, but is not currently available [27]. Acquiring this knowledge can be very useful for designing packaging labels that are meaningful and relevant for consumers. Moreover, this knowledge may also be of interest for loose fruit commercializers to provide consumers with the most relevant information. It can also help to adequately supply information online, as online grocery shopping is predicted to continue to increase [11]. 

Different studies have highlighted the dominance of hedonic attributes in consumer food choices [5,24,28], which is supported by the marked effect that fruit sensory properties had on consumer choice and purchase intention [4,6,29]. In this context, there is an increasing trend among fruit distributors to include sensory claims on fruit packaging. Although only one study has approached the effect of sensory claims on consumer fruit choice to date, it demonstrated a significant effect [29]. So, in a few years, sensory labels on fruit packaging will likely become habitual to help consumers predict to what extent the product matches their preferences. So, it is necessary to identify those sensory attributes that consumers need to know in accordance with fruit type because, according to sensory studies, the relevance of different attributes on consumer preferences depends on fruit [2,3,4,30,31,32,33,34].

In this context, this study approaches three objectives: (1) Determine if consumer fruit choice (packaged vs. loose fruit) is conditioned by their information needs; (2) identify information gaps on fruit packaging labels; (3) establish the key attributes for sensory label designs depending on fruit type. 

## 2. Materials and Methods 

Three hundred and ninety-four Spanish consumers participated in this study, which is based on an online questionnaire via the online platform Google forms (www.googleforms.com). The participants were recruited using the consumer database of the Sensory and Consumer Science research group from the IVIA, Valencia (Spain). Only those people who buy fruit at least once a week were invited to participate, and all of them signed an informed consent form. 

The questionnaire was arranged into three parts: 

1. In the first section, the participants were asked ‘What kind of fruit do you normally buy?’ They had to choose among three options: ‘I usually buy packaged fruit’, ‘I usually buy loose fruit’, ‘I usually buy both types: Packaged and loose fruit’.

Then they were asked about their information needs at the time they bought fruit: “When you purchase fruit, are you interested in receiving information about it?” Response options: Yes/No.

The people who stated their interest in information were asked: “Does your purchase decision depend on the information you have received?” Response options: Yes/No/Depending on the day.

2. In the second section, we investigated the information gaps on fruit packaging labels. To this end, the consumers who had indicated they bought packaged fruit were provided with a list of labels and were instructed to check all labels they were usually provided with when purchasing packaged fruit. 

Then the same list was provided to the participants together with the instruction ‘Check all the labels that you would like to receive when you buy fruit.’ 

In both cases, the list of labels included: Name of the fruit; Company (brand); Variety; Country of origin; Production area (region, state); Size; Category (premium, extra, first); Batch number; Presence/absence of seeds (e.g., citrus fruits, watermelon); Treatments applied after harvest; Taste, aroma, and texture characteristics that are perceived when eaten; Recommended use (e.g., ideal for juice, to add to salads, etc.); Nutritional value; Production method (e.g., eco, bio, etc.); Preservation method; Preparation recommendations (e.g., wash before consume, consume at room temperature, etc.); Quality Seals (PDO, PGI, etc.); Best before date; Environmental information (e.g., if it is locally produced); Beneficial properties (e.g., rich in fiber, high content in vitamin C); If it is “Ready to eat”; Net weight; Number of pieces; Packing date; % of the price received by the farmer; Harvest date; None of the above. 

To draw up the labels list, individual interviews were held with eight people (4 men and 4 women aged 18–57 years) who stated they eat fruit at least once a week. Consumers were asked to first list all the information they remembered they were provided with when they bought fresh fruit, and then include on the list that information they would like to know, but is not normally provided.

The final labels list was drawn up after taking into account the information that must be mandatorily provided, the results of the individual interviews, and the evaluations of fruit packaging in supermarkets. The option ‘Others’ was also available if some participants wished to indicate any information missing on the list. Before conducting the final questionnaire, it was individually checked with six people in order be sure that all the vocabulary and concepts could be understood by regular consumers and no relevant information was missing.

3. Finally, those participants who declared an interest in “Taste, aroma, and texture characteristics that are perceived when eaten”, were moved to the third questionnaire section.

In this part, the participants were presented images of six different fruit types and were asked about the organoleptic characteristics they would like to know when they bought fruit. The different fruit types were shown in six pictures, which included: (1) Citrus fruit (mandarins and oranges); (2) stone fruit (plums, apricots, peaches, nectarines); (3) pome fruit (different pear and apple varieties); (4) melon and watermelon; (5) different types of bananas; and (6) kiwi (yellow and green kiwis). 

A preliminary sensory attributes list was drawn up based on a fruit sensory studies bibliography [3,4,35,36]. The final list was design with the help of six consumers, who added missing terms after check the list and the fruit images. Hence, the final list included ‘easiness to peel’, ‘astringency level’, ‘mealiness’, ‘crunchiness’, ‘firmness’, ‘juiciness’, ‘sweetness’, ‘sourness’, ‘taste intensity’, ‘seed content’, ‘aroma intensity’, ‘none of them, it is enough with the aspect’, and ‘others’. They were also given the option to check ‘I don’t usually buy this fruit type’.

Participants were presented the final list and asked to check all the attributes they would like to know when they purchase the different fruit types shown in the images. The order the images were presented in was randomized, as were the terms on the lists [37].

At the end of the questionnaire, the participants answered some demographic questions, such as their fruit consumption frequency, gender, and age (18–25 years old, 26–40 years old, 41–55 years old, and older than 55 years old). 

Packaged fruit in Spain is mainly sold in supermarkets, and suppliers normally provide consumers with more information than is mandatory, often for marketing purposes. Thus, in order to know what kind of non-mandatory information is normally included on package labels, we performed a prospective study by visiting 10 supermarkets belonging to five main chains. This allowed us to collect in situ all the information provided on fruit packaging to gain the main insights received by consumers. Examinations were made of the principal fruits herein included. 

## 3. Results and Discussion

### 3.1. Consumer Interest in Information

**Figure 1:**
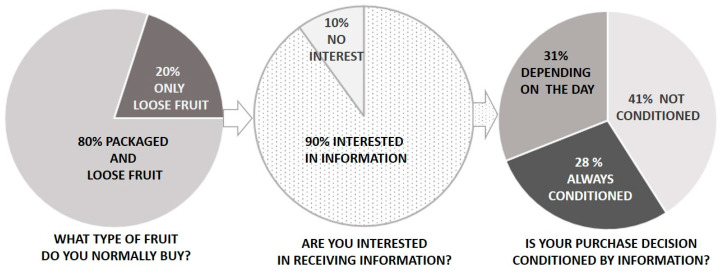
Consumer purchase practices and interests in information. Consumers response to different questions about purchase habits and information interest.

The first objective of this study was to evaluate consumer interest in fruit information and to determine if it conditions fruit selections (packaged vs. loose fruit). To this end, we asked the participants about their usual way of buying fruit and their interest in receiving information about it. Our results showed that for 80% of the participants, fruit purchases habitually include both packaged and loose fruit, while the other 20% only bought loose fruit (Figure 1). 

Our data revealed that for both consumer groups, the same percentage of them (89.82% for loose fruit, and 89.84% for packaged fruit) were interested in receiving information about fruit when they purchase. So, despite packaging labels being a relevant source of information, consumer information needs were revealed to be a non-decisive factor for deciding to choose packaged or loose fruit. 

When we took the question a step further and asked the participants if their purchase decisions depended on labeled information, we found that not all consumers were interested in the provided information to base their final decisions on it. According to our data, 41% of people who stated they were interested in information do not use the information they receive for purchase decisions. This result shows that for a high percentage of consumers, labels do not play an important role in their fruit purchases.

Of the 59% participants who affirmed that their decisions were conditioned by label information, 28% declared that this was always the case, while 31% stated that information influence depended on the day (Figure 1). Therefore, our results revealed a gap existed between information interest and information use, i.e., deciding fruit purchases based on the received information. Different studies have reported that too much information on labels can lead consumers to not assimilate it, mainly because time is a limiting factor while shopping [13,20,24]. Therefore, today’s busy lifestyle and lack of clear labels are likely the reasons why the extent of information influence (on those consumers who declared being interested) depended on the day. 

Our results also showed that a higher percentage of females (62% of the female participants) than males (54% of the male participants) based their purchase decisions on available information. Moreover, the effect of label cues on choice decisions was stronger for the participants aged over 40 years than among younger participants. Accordingly, it has been reported that women spend more time reading food labels than men, as do older people [13,38], which may be related to their greater concern about health and sustainability [13,21,39]. In line with this, Galati et al., 2019 [40] have reported that one of the main reasons for increasing consumer information needs is to avoid their confusion and uncertainty about the impact of food consumption on human health and the environment. 

### 3.2. Identification of Information Gaps 

Information provided to consumers is effective only when it addresses specific information needs, matches certain interests, and can be processed and used by its target audience. Hence, insights into consumer needs for, and interest in, information are required before needs can be effectively addressed [41]. If we bear this in mind, this study explored the information gaps associated with packaged fruit commercialization. Information gaps can be defined as that information which consumers need to know to make fully-informed decisions, but is not available. Thus, in this part of the study, only the data from the people who stated being interested in information and in habitually purchasing packaged fruit (283 participants, 72% of all the participants) were taken into account (Table 1).

A 20% difference between those consumers who declared they wished to receive a specific label and those who stated having received was established as the threshold to identify gaps. It is important to clarify that the fact that the participants who declared not receiving specific information does not necessarily imply that it was left as not provided. Perhaps a certain number of responses in this regard came from people who received information, but did not assimilate it. These cases form part of the information gap concept, as it is assumed that the information which consumers stated they are interested in could not be processed and used because it was not properly conveyed. However, to gain a clearer view about this, while this study was underway, we visited 10 supermarkets to detect first-hand the non-mandatory labels that are habitually included on fruit packaging. The obtained results are discussed together with consumer data. 

As seen in the right column of Table 1, when the packaged-fruit consumers were asked to indicate all the labels that they would like to be provided with on packaging, fruit origin information (including country of origin and production area labels) and production method were the labels that they found most interesting. This result falls in line with Gao et al., 2014 [6], who reported that not only physical attributes, but also these two credence attributes (fruit origin and production method), strongly impacted consumer fresh fruit choices, which were closely related to consumer fruit quality perceptions. Moreover, the marked consumer interest in knowing the fruit production area herein detected falls in line with previous research works that have reported production area to be a decisive factor for purchase decision making. A growing popularity of locally produced food has been reported [42], and several drivers of consumers’ local food choices have been identified: Benefits beyond self-interest with advantages for society, environmental and sustainable food policy development [43]; intrinsic product quality; local support and provenance [44].

Of the three labels herein identified as the most interesting ones for consumers (country of origin, production area, and production method), the only one that must be mandatorily included on packaging is the country of origin [45]. However, we corroborated in supermarkets that production area was habitually provided, mainly for national fruit. For production method, we found that four of the five supermarket chains offered ‘organic fruit’, but this was the only available information about production method. In fact, in this study, it was identified as one of the information gaps.

Fruit name and variety, together with labels associated with fruit freshness (packing date, harvest date, best before date), were also relevant for a high percentage of consumers (60–75%). Within the same range of importance, we found information about the percentage of price perceived by farmers, quality seals, and net weight. Of all these labels, three must be included on packaging: Fruit name, variety, and net weight (fruit name only if product content cannot be seen) [45]. Two major gaps were identified in this group of labels, as more than 60% of consumers were interested in knowing information that is never currently provided: Harvest date and percentage of price perceived by farmers. 

Consumers’ need to know the fruit harvest date before purchase is related to the “freshness” concept. Indeed, freshness has been reported as one of the main drivers for consumer choices of different fruit [46,47]. Regarding the percentage of price perceived by farmers, this label need is in accordance with consumers’ increasing interest in “fair trade” products [23,48,49]. In addition, the recent lockdown associated with Covid-19 highlighted the key role that farmers play in supplying basic food to society and opens debate about the pressure that commercial chains exert on farmers. Consequently, Spanish consumers are becoming more aware of the need for farmers to be paid fair prices for their products.

Information on postharvest treatments, environmental aspects, organoleptic attributes and nutritional values were interesting for 50–60% of the participants, and were all identified as major information gaps. Consumer interest in the environmental-related aspects herein detected agrees with previous studies, which have reported a growing consumer awareness of the environmental impact of their purchasing decisions [50]. Accordingly, in a recent study we detected that plastic packaging was one Spanish consumer barrier to buying fresh cut fruit [51]. Consumers’ plastic overuse concern is likely to be one of the reasons for consumer needs for environmental information. 

In line with this, our data showed that all the participants declared buying loose fruit to a greater or lesser extent (20% of consumers declared purchasing only loose fruit, while 80% declared buying both loose fruit and packaged fruit). However, no consumers reported that they based their fruit purchase on only packaged fruit (Figure 1). Therefore, it is likely that consumer choice of loose fruit is related to increasing environmental concerns, as loose fruit offers the advantage of reducing plastic overuse. However, in a recent review, White and Lockyer (2020) [11] highlighted the need to consider the potential increase in food waste that would be associated with a drastic removal of plastic packaging from fruit and vegetables. According to these authors, food waste linked with plastic package removal for fresh fruit and vegetables is likely to have an even stronger environmental impact than producing and disposing of plastic. However, use of packaging is not always justified. For example, packaging is needed to extend the postharvest life of very susceptible fruit to mechanical damage and spoilage, like small berries [52,53], but can be avoided with other not so fragile fruit. Oranges, pomegranates, or bananas are good examples of fruit whose packaging step can be eliminated, which would have no marked effect on shelf life. 

In this context, the food industry is making an effort to find new alternative materials to plastic, such as biodegradable film and other materials like biocomposite, which preserve product quality, but cut the use of non-renewable resources and prevent plastic waste from accumulating [54,55]. 

Similarly, consumer awareness of the food impact on health has considerably increased in the last few decades [23], and concerns about food safety, produce quality, and pesticide abuse have been identified as the main factors that motivate consumers’ willingness to pay a premium for eco-labels, organic labels, and pesticide-free labels [6].

The environmental, nutritional, and organoleptic-information gaps revealed by consumer data coincided with the reality perceived in supermarkets. That is, the only mandatory information in this regard is to specify the postharvest treatments applied to citrus fruit, but not to other fruit [56]. A common trend detected in all supermarkets for all fruit was the presence of information that intended to highlight sustainable packaging (80% reduction in plastic, recycled material, etc.), which reflects the clear intention to respond to consumer needs. However, it was exceptional finding information related to other sustainability and health aspects, like “pesticides free” or “local produce” labels, and no references appeared about aspects like “rational use of water”, “CO_2_ footprint”, etc. Our observations also indicated that providing nutritional labels was quite unusual. 

With regard to sensory attributes, it has been reported that consumer decisions may be modified by providing fruit sensory label [29]. When sensory information is lacking, consumer expectations of a fruit pre-purchase situation are based mainly on: (1) Fruit appearance; or (2) their previous experience with that specific product. However, it has been demonstrated that fruit appearance may be a poor indicator of fruit internal quality [57]. Moreover, there are different purchase situations in which previous experience may not exist, when buying fruit for the first time in a new market, and also in a specific market because distributors may change throughout the season depending on the supply/demand. Thus, sensory labels may become key factors for consumer predictions of how much a product matches their preferences. 

Of the five supermarket chains that we visited, three were highlighted for providing sensory labels, but they were restricted to only a few types of fruits, mainly apples, pears, grapes, and pineapple. Most claims referred to only one attribute, generally level of sweetness. Only in apples did labels cover attributes related to different properties, like flavor (sweetness, acidity), texture, and aroma. Availability of such sensory labels may allow consumers to make easier selections from among apple varieties.

Finally, four gaps were identified among the labels that aroused the interest of less than 50% of consumers: ‘Preservation method’, ‘beneficial properties for health’, ‘if it is ready to eat’, and ‘preparation methods’. Indeed, we corroborated that such information was generally lacking in supermarkets, and recommendations about preservation method were exceptional and limited to storage temperature. Claims related to beneficial properties were scarce and linked with only the high vitamin C and fiber content of kiwi and coconut. Preparation recommendation was available only for big-sized fruit like pineapple, melon and watermelon, and only one claim that referred to the ‘ready to eat’ state of pineapples was identified.

It is also interesting to highlight that the opposite happened with ‘brand’ as 73% of consumers were aware of finding this information on labels, but only 43% of them were interested in it. According to this result, brand name should not occupy a priority position on labels, which should be left for other more relevant information for consumers so that it can be more clearly visualized.

However, we must keep in mind the importance of information for food safety that is not very interesting for consumers. Thus, food traceability linked with batch numbers is key to avoid public health risks. Nowadays, information behind batch numbers or barcodes is not generally accessible for consumers. Quick Response Codes (QR codes) have been reported as useful tools to not only save information traceability, but also provide consumers with further information than that printed on packaging [58,59,60]. QR codes allow consumers easy access, by means of smartphones, to information about the item to which it is attached. These optical labels have a greater storage capacity compared to standard barcodes. This means that QR codes are interesting tools to provide consumers with information that is apparently not so relevant for them, while maintaining on packaging those labels that are more decisive for purchase decisions. QR codes can be very useful for providing information that is essential for only a few consumers, for example, those people with food intolerance, and would extend nutritional labels [61].

### 3.3. Sensory Labels Design Depending on Fruit Type

According to ASTM International (2016) [62], a sensory label is a ‘statement about a product that highlights its advantages, sensory or perceptual attributes, or product changes or differences compared to other products in order to enhance its marketability’. Sensory labels are categorized into ‘comparative’ and ‘non comparative’ labels [62]. Comparative claims compare similarities and differences between two products or more (different brands or formulations/recipes in the same brand). Non-comparative labels convey something specific about a single product in terms of its characteristics or performance. While objective attributes like ‘very sweet’ are relatively easy to substantiate, more subjective characteristics of product or product experience like ‘delightful fruit flavor’ or ‘natural taste’ are more difficult to substantiate. This study focused on identifying those objective attributes that are of particular interest for consumer choice decisions among those that confer the sensory profile of different fruit to obtain an appropriate sensory label design. 

**Figure 2:**
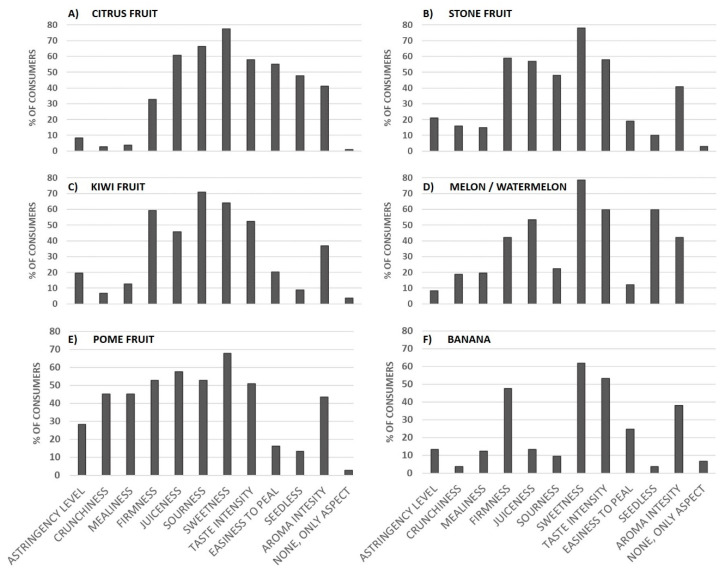
Sensory label content depending on the fruit type: (**A**) citrus fruit; (**B**) stone fruit; (**C**) kiwi fruit; (**D**) melon/ watermelon; (**E**) pome fruit; (**F**) banana. Percentage of consumers (among those interested in receiving information and who normally purchase packaged fruit) who declared being interested in acquiring information about different sensory attributes for several fruit types.

Figure 2 represents, for the main different fruit types, the percentage of consumers who wish to receive information about the different sensory attributes associated with this foodstuff type. The results showed that there were two especially relevant sensory attributes as consumers wished to receive information about them regardless of fruit type. These two attributes were related to flavor, specifically sweetness level and taste intensity, which were chosen in all cases by 50–80% of the participants (Figure 2A–F). Of these, sweetness seemed the most relevant characteristic for consumers to know because it was chosen by more than 60% of the participants irrespectively of fruit type. 

Another flavor-related attribute, level of sourness, was also pointed out as key information for facilitating consumer choice decisions with citrus or kiwi fruit (Figure 2A,C). It was also relatively important for pome and stone fruit (Figure 2B,E), but less relevant for bananas and melons/watermelons (Figure 2D,F). 

The identification of sweetness as a sensory attribute of special relevance is in accordance with that reported in sensory studies. In such studies, consumer preferences and purchase intentions after tasting fruit were related to fruit sensory properties, and in such a way that the attributes which acted as drivers of liking were identified. Thus, sweetness has been reported to be a driver of liking for pome fruit, such as apples and pears [35,63], stone fruit like nectarines and peaches [36,64], or kiwis [65], mandarins [4,66], bananas [67], and melons [68]. 

Our results related to sourness fell in line with those reported by Jaeger et al. (2011) [69] and Tarancón et al. (2020) [4], who found the sourness level to be a determinant for consumers liking kiwi and citrus fruit (mandarins). 

As can be observed in Figure 2B,E, the attributes associated with texture properties were also important for consumers who were especially interested in receiving information about juiciness and firmness of pome and stone fruit. Juiciness was also relevant for citrus fruit, and to a lesser extent for melons/watermelons. Firmness was also pointed out as a main kiwi attribute (Figure 2C). Consumer information needs related to texture characteristics are clearly linked with the relevance that these attributes have for the sensory acceptance of such fruit [4,35,36,62,65,68].

Other texture attributes, such as mealiness and crunchiness, were selected only for pome fruit (between 30–45% of consumers) (Figure 2E), while astringency level was the least chosen attribute, and was only somewhat significant for stone and pome fruit (Figure 2B,C). Our results agree with previous studies that have reported mealiness, crunchiness, and astringency levels as determinant attributes for consumers liking stone and pome fruit [3,70,71].

Aroma intensity was selected for all the fruit types, but only for 40% of consumers. This data indicates that aroma properties are less relevant for consumer liking expectations than flavor or texture attributes.

Finally, aspects as to whether fruit being easy to peel or containing seeds were only significant for citrus and watermelons/melons, and the latter only for seeds (Figure 2A,D). The importance of seed content in citrus fruit has been previously reported in studies conducted with consumers by Tarancón et al. (2020) [4]. In fact, Commission Regulation (EU) No. 543/2011 [56] establishes the obligation to include on labels the indication ‘seedless’ for seedless clementines (no seeds) and ‘with seeds’ for clementines with more than 10 seeds.

The information herein collected is very useful for a sensory label design that responds to consumer information needs. However, it is important to bear in mind that during the shelf-life period and home storage, fruit may undergo metabolic changes that lead to sensory modification in relation to harvest time [72]. Thus, the sensory information customers are provided with should be linked mainly with characteristics intrinsic to variety. In this way, in a pre-purchase situation, consumers would be able to know if a specific variety matches their preferences. Moreover, providing information about sensory attributes linked with fruit maturity may be of special interest for non-climacteric fruit whose maturity barely evolves after harvest [72]. For example, the acidity level of citrus fruit markedly lowers from the beginning to the end of the harvesting season [73], which is more significant than the slight changes that may occur during shelf life [57]. Therefore, in such situations, updating sensory label information throughout seasons may be particularly interesting for consumers. 

## 4. Conclusions

Based on our results, we conclude that Spanish consumers are very interested in receiving information when they purchase fruit. However, their choice between packaged and loose fruit does not depend on their information needs. In fact, a gap between information interest and information use was detected as their final purchase decisions are not always based on the provided information. ‘Harvest date’, ‘production method’, ‘percentage of the price received by farmers’, ‘postharvest treatments’, ‘sensory properties’, and ‘environmental information’ were identified as the major information gaps and, therefore, the industry should make efforts to provide this information or make it clearer than it currently is. For a correct design of sensory labels, they should include information on ‘sweetness’ and ‘flavor intensity’ irrespectively of fruit type. ‘Sourness’ and ‘juiciness’ levels must be included on citrus fruit labels, while ‘sourness’ and ‘firmness’ are relevant for kiwis. Information on texture properties must be provided to help consumers choose pome and stone fruit. Other attributes, such as easiness to peel, are important only for citrus fruit.

## Figures and Tables

**Table 1:** Information gaps. Percentage of consumers who declared receiving different packaging labels when purchasing fruit vs. the percentage of those interested in each one. A 20% difference was established as the threshold to identify gaps, which are indicated with **.

Labels	% of Consumers	
	Receive	Wish to Receive
Production area	75.3	84.4
Country of origin *	84.8	82.7
Production method	41.7	79.1 **
Name of fruit *	91.5	74.2
Variety *	64.7	66.8
Packing date	68.9	66.8
Harvest date	0.3	62.9 **
% of the price received by the farmer	0	61.5 **
Quality Seals	42.0	61.1
Best before date	52.3	60.8
Net weight *	70.3	60.4
^$^ Application of treatments	4.9	54.1 **
Taste, aroma, and texturecharacteristics	4.6	52.3 **
Environmental information	13.1	51.6 **
Nutritional facts	23.7	51.2 **
Category/Class *	46.6	48.1
Preservation method	19.4	47.0 **
Company (Brand) *	73.1	42.8
Beneficial properties	11.7	40.3 **
If it is “Ready-to-eat”	13.1	37.1 **
Preparation recommendations	12.4	36.4 **
^$^ With seeds or seedless	22.3	33.6
Recommended use	15.9	32.9
Number of pieces *	27.2	30.7
Size (caliber) *	41.7	30.0
Batch number *	57.2	26.9
None	0.7	-

* Indicates that this information is mandatory to be provided irrespectively of fruit. ^$^ indicates information that must be provided only for citrus fruit.

## Data Availability

The data presented in this study are available on request from the corresponding author.
